# Comparative evaluation of immunoserological detection of F-actin antibodies

**DOI:** 10.1371/journal.pone.0345250

**Published:** 2026-04-07

**Authors:** Friederike Dellbrügge, Stephanie Loges, Nicole Henjes, Heiner Wedemeyer, Richard Taubert, Bastian Engel

**Affiliations:** 1 Department of Gastroenterology, Hepatology, Infectious Diseases and Endocrinology, Hannover Medical School, Hannover, Germany; 2 Member of the European Reference Network for Hepatological Diseases (ERN RARE-LIVER), Hamburg, Germany; 3 PRACTIS Clinician Scientist Program and CORE100Pilot Clinician Scientist Program, Dean’s Office for Academic Career Development, Hannover Medical School, Hannover, Germany; Medizinische Fakultat der RWTH Aachen, GERMANY

## Abstract

Autoantibody detection remains essential in diagnosing autoimmune liver diseases (AILD), particularly autoimmune hepatitis (AIH), in which smooth muscle antibodies (SMA) targeting filamentous actin (F-actin) are commonly observed. This study assessed the concordance between immunofluorescence testing (IFT), the gold standard for SMA detection, and a commercially available F-actin ELISA in a real-world clinical setting. During the three-months study period, 244 samples were sent to our laboratory for autoantibody testing; 241 were included in the study. SMA IFT on rodent stomach, kidney and liver sections identified 104 positive cases, with ELISA-detected F-actin antibody levels being significantly higher in these cases. F-actin ELISA concentrations significantly increased with higher SMA IFT titer. Concordance between the ELISA (using manufacturer-recommended cut-offs of 20 and 30 units) and IFT depended on the SMA staining pattern. Only slight agreement was found between positive kidney SMA staining patterns and ELISA concentration. Stomach SMA staining showed moderate agreement (Cohen’s kappa = 0.49) and a subgroup analysis revealed better agreement in autoimmune disease cases, particularly AIH. An optimized ELISA cut-off of 27.6 units improved agreement slightly (Cohen’s kappa = 0.55). The results suggest that, although there is a certain level of concordance between the ELISA and stomach SMA IFT, the F-actin ELISA does not reliably predict SMA IFT staining. These findings underscore the importance of method validation and context-specific interpretation when implementing ELISA-based autoantibody screening in routine diagnostics.

## Introduction

Screening for the presence of autoantibodies is a mainstay in the work-up of any patient with a non-viral liver disease. The presence of autoantibodies is prominently acknowledged in current guidelines for the diagnosis of autoimmune liver diseases (AILD), mainly Autoimmune Hepatitis (AIH) and Primary Biliary Cholangitis (PBC). While antimitochondrial antibodies (AMA) are pathognomonic for the latter, the former is a rare AILD that lacks specific pathognomonic features [1]. This poses a significant diagnostic challenge and the diagnosis is obtained using a scoring system that incorporates different clinical features including the presence of autoantibodies [2–4]. Antinuclear autoantibodies (ANAs), smooth muscle antibodies (SMA), liver-kidney microsomal antibodies (LKM), liver cytosol type 1 antibodies (LC1), and soluble-liver-antigen/liver pancreas antibodies (SLA/LP) are detected in AIH patients, but especially ANA and SMA lack specificity while LKM, LC1 and SLA/LP lack sensitivity [2–5].

SMA target a variety of smooth muscle antigens and are considered a phenotypical autoantibody in AIH [6,7]. One of the main antigenic targets for SMA at the protein level is filamentous actin (F-actin) [8–10]. F-actin is essential for the structure of smooth muscle and consists of strung together globular actin (G-actin) molecules forming a chain-like structure. F-actin antibodies are specific to F-actin most likely due to targeting conformational epitopes of F-actin rather than the single G-actin molecule [8,11,12]. SMA immunofluorescence staining patterns can be found on kidney tissue (e.g., as staining of vessel (V), glomeruli (G) and/or tubules (T)), liver cells as well as the muscularis mucosae of the gastric wall of rodent tissue substrates [13].

Screening for autoantibodies using immunofluorescence testing (IFT) on rodent substrates for ANA, SMA, LKM and LC1 is recommended as the gold standard by the international AIH group and the current international guidelines [7,14–16]. However, the technique requires highly qualified staff for staining and assessment making IFT autoantibody screening costly and limited to specialized centers. Commercial ELISAs offer a cheaper, more accessible alternative with potentially lower variation between different observers that may be automated in high-throughput laboratories. For F-actin there is one CE-marked ELISA commercially available using F-actin as the target antigen which recently demonstrated good diagnostic performance to predict AIH [4].

Yet, considerable differences between inter-rater reliability of IFT and commercial ELISAs depending on the manufacturer for ANA and LKM1 were highlighted in previous studies [17,18]. In these studies inter-rater reliability could be improved by adjusting ELISA cut-offs using local cohorts for validation [18].

As comparable data is missing on the concordance of a F-Actin specific ELISA and IFT as the gold standard that could inform decisions in real-world laboratory practice, this study aimed to investigate the concordance between the commercially available F-actin ELISA and SMA IFT.

## Materials and methods

### Study population

In this monocentric study we collected data from adult (age ≥ 18 years) patients that were evaluated for the presence of autoantibodies with both IFT (rat liver, kidney and stomach) and F-actin ELISA between January 15th 2024 and March 15th 2024 at Hannover Medical School (MHH), Hannover, Germany. Autoantibody testing was ordered by the respective treating physicians. All tests were performed in our autoantibody laboratory as part of the routine laboratory work (Institute of Clinical Chemistry and Central Laboratory, MHH, Germany). Patients receiving only ELISA or IFT were excluded. The data was obtained from clinical records between the 10th and 12th December 2024 and anonymized directly after data collection. Laboratory test results including aspartate aminotransferase (AST), alanine aminotransferase (ALT), bilirubin, albumin, immunoglobulin G (IgG) and IgM were obtained from medical records at the time of autoantibody testing. Data were collected retrospectively and analyzed after de-identification.

### IFT staining

IFT staining was performed using a commercially available rodent multi-organ substrate panel (LKS Rat wrapped Standard Kit, Aesku.Diagnostics GmbG & Co., Wendelsheim, Germany) according to the manufacturer’s instructions. Patient serum samples were diluted starting from 1:20 up to 1:160. Antibody staining patterns were evaluated and interpreted according to current guidelines using a fluorescence microscope by two experienced observers (NH, SL) (Olympus BX60 Microscope, Evident Europe GmbH, Hamburg, Germany) [14].

### F-actin ELISA

For F-actin antibody detection a commercially available ELISA assay (QUANTA Lite Actin IgG, Werfen, Germany, ref: 708785) detecting IgG antibodies against filamentous actin was used. F-actin ELISA assays were performed according to the manufacturer’s protocol. Technical replicates of two were performed for each sample and the mean of both was used for further analysis. Observers were blinded to clinical information.

### Statistical analysis

Statistical analysis was performed using R Statistical Software (version 4.2.2, R Core Team). The inter-rater reliability of stomach SMA IFT and F-actin ELISA was assessed by the Cohen’s kappa coefficient (k) using the “DescTools” package. Kappa values were considered as almost perfect agreement (>0.80), substantial agreement (0.61–0.80), moderate agreement (0.41–0.60) and slight agreement (<0.40) according to the Landis-Koch scale [19]. An optimized cut-off for the F-actin ELISA assay was assessed with IFT staining as standard using the “cutpointr” package to calculate the maximized Youden function.

To calculate p-values we used the Mann–Whitney U test for two continuous variables and the Chi^2^ test for categorical variables. For p-value calculation of more than two continuous variables the Wilcoxon rank sum test with Benjamini-Hochberg correction was used. Continuous variables were presented with median and IQR. For categorical variables we used absolute numbers and percentages. A p-value of < 0.05 was considered statistically significant.

### Ethics

This study was performed according to the 1975 Declaration of Helsinki and approved by the local Ethics Committee (protocol numbers 2817-2015 and 11255_BO_K_2024, MHH Ethikkommission, Hannover, Germany). Since the data was anonymized before analysis and data was collected exclusively from existing clinical data no written informed consent was required.

## Results

### Patient cohort

**Fig 1:**
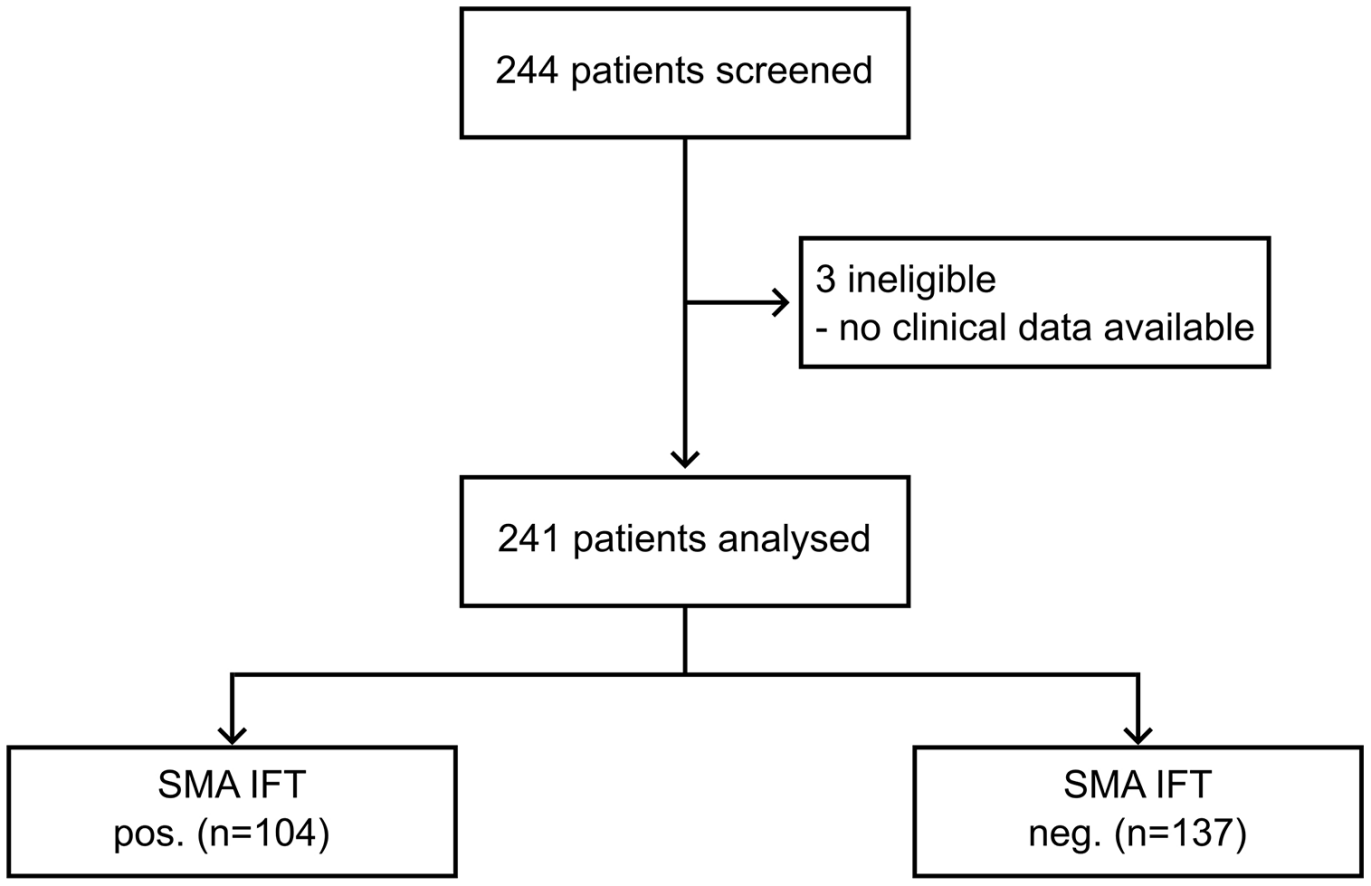
Flow chart of patient selection. IFT: immunofluorescence testing. Positivity was defined as a positive SMA signal on rat stomach or kidney tissue at a dilution of 1:40 or higher.

Two-hundred forty-four samples were sent for assessment of autoantibodies by IFT and F-Actin ELISA between 15.01.2024 and 15.03.2024 at Hannover Medical School (Hannover, Germany). Of these, three samples could not be connected to clinical data. Hence, 241 samples were eligible for analysis in this study (Fig 1).

IFT was regarded as the reference method and samples were stratified by IFT SMA titer ranging from negative to 1:160. One-hundred and four samples had a titer of 1:40 or higher and were therefore considered positive for SMA by IFT. One-hundred thirty-seven samples were SMA IFT negative. Samples that were positive for SMA on IFT had higher F-actin ELISA levels and higher IgG levels (Table 1).

**Table 1:** Demographic and clinical features of the cohort differentiated by IFT.

Variable	SMA negative in IFT	SMA positive in IFT	p
**n**	137	104	
**Age [years]**	53 [37, 62]	54.5 [37, 64]	0.477
**Sex = male (%)**	62 (45.6)	60 (57.7)	0.084
**ALT [U/l]**	45 [26, 86]	41 [26, 99]	0.904
**AST [U/l]**	35 [26, 74]	45 [38, 98]	0.063
**AP [U/l]**	122 [77 195]	116 [81, 184]	0.907
**yGT [U/l]**	69 [32, 174]	88 [34, 202]	0.637
**Bilirubin [µmol/l]**	11 [7.00, 28]	16 [9, 35]	0.057
**Albumin [g/l]**	40.00 [33, 44]	39 [32, 44]	0.361
**IgM [g/l]**	1.28 [0.86, 2.13]	1.27 [0.84, 2.07]	0.831
**IgG [g/l]**	12.34 [10.04, 15.22]	13.82 [11.20, 18.92]	0.002
**F-actin ELISA [units] (median [IQR])**	11.00 [6.10, 18.40]	15.95 [10.38, 34.20]	<0.001
**Diagnoses (%)**			
**AIH**	9 (6.6)	10 (9.6)	
**Drug-induced autoimmune-like hepatitis**	1 (0.7)	0 (0.0)	
**Cryptogenic liver disease**	21 (15.3)	17 (16.3)	
**DILI**	4 (2.9)	1 (1.0)	
**Patients after liver transplantation**	8 (5.8)	2 (1.9)	
**Non-autoimmune liver disease**	51 (37.2)	49 (47.1)	
**PBC**	15 (10.9)	1 (1.0)	
**PBC/AIH overlap**	3 (2.2)	1 (1.0)	
**PSC**	6 (4.4)	7 (6.7)	
**PSC/AIH overlap**	0 (0.0)	3 (2.9)	
**Systemic autoimmune disease with liver involvement**	2 (1.5)	1 (1.0)	
**Systemic autoimmune disease without liver involvement**	7 (5.1)	2 (1.9)	
**No liver or systemic autoimmune disease**	10 (7.3)	10 (9.6)	

Continuous variables are presented as median (IQR) and categorical variables are presented as absolute number (frequency in percent). P-values were calculated with the Mann–Whitney U test for continuous variables and the Chi^2^ test for categorical variables. ALT: alanine aminotransferase; AST: aspartate aminotransferase; AP: alkaline phosphatase; yGT: gamma-glutamyl transferase; IgM: immunoglobulin M; IgG: immunoglobulin G; F-actin: filamentous actin; AIH: autoimmune hepatitis; DILI: drug-induced liver injury; PBC: primary biliary cholangitis; PSC: primary sclerosing cholangitis.

Sex, age as well as other clinical or laboratory parameters did not differ between groups. There were no major differences in the frequency of respective diagnoses. Twenty-four AIH cases (including overlap patients) were represented in this study and divided equally between both groups (Table 1). The frequency of SMA staining patterns varied. SMA staining of kidney vessels was observed in 96 patients, whereas staining of glomeruli or tubules was detected in only 28 patients. Additionally, 53 patients showed positive SMA staining in the muscularis mucosae of the stomach.

The manufacturer recommends two cut-off values for the F-actin ELISA with 20 units for moderate positivity and 30 units for high positivity. Seventy-one samples were considered positive with 170 negative samples when using the cut-off of 20 units. At the cut-off of 30 units 52 samples were considered positive and 189 samples negative for F-actin. For both cut-offs age and sex did not differ between negative and positive samples. Samples positive for F-actin at both cut-offs showed higher aspartate aminotransferase, higher alanine aminotransferase, higher alkaline phosphatase, higher gamma-glutamyl transferase, higher bilirubin, higher immunoglobulin G, higher immunoglobulin M and lower albumin levels (Tables 2 and 3).

**Table 2:** Demographic and clinical features of the cohort differentiated by ELISA cut-off 20.

Variable	F-actin ELISA cut-off 20 units		
	F-actin negative by ELISA	F-actin positive by ELISA	p
**n**	170	71	
**Age [years]**	53.00 [37.00, 63.00]	54.00 [38.50, 64.00]	0.512
**Sex = male (%)**	91 (53.8)	31 (43.7)	0.194
**ALT [U/l]**	38.00 [24.00, 82.00]	52.00 [32.00, 117.25]	0.019
**AST [U/l]**	35.00 [25.25, 66.25]	57.50 [33.00, 141.00]	<0.001
**AP [U/l]**	106.50 [70.00, 175.25]	136.00 [104.50, 209.75]	0.004
**yGT [U/l]**	67.00 [29.25, 146.00]	111.00 [42.25, 243.50]	0.013
**Bilirubin [µmol/l]**	11.00 [7.00, 24.50]	21.50 [9.00, 60.50]	0.008
**Albumin [g/l]**	41.00 [34.50, 44.00]	36.50 [28.75, 41.00]	<0.001
**IgM [g/l]**	1.20 [0.76, 2.00]	1.43 [1.10, 2.20]	0.025
**IgG [g/l]**	12.30 [9.95, 14.49]	16.24 [12.61, 20.93]	<0.001
**Diagnoses (%)**			0.002
**AIH**	7 (4.1)	12 (16.9)	
**Drug-induced autoimmune-like hepatitis**	1 (0.6)	0 (0.0)	
**Cryptogenic liver disease**	30 (17.6)	8 (11.3)	
**DILI**	4 (2.4)	1 (1.4)	
**Patients after liver transplantation**	7 (4.1)	3 (4.2)	
**Non-autoimmune liver disease**	74 (43.5)	26 (36.6)	
**PBC**	9 (5.3)	7 (9.9)	
**PBC/AIH overlap**	1 (0.6)	3 (4.2)	
**PSC**	10 (5.9)	3 (4.2)	
**PSC/AIH overlap**	0 (0.0)	3 (4.2)	
**Systemic autoimmune disease with liver involvement**	2 (1.2)	1 (1.4)	
**Systemic autoimmune disease without liver involvement**	6 (3.5)	3 (4.2)	
**No liver or systemic autoimmune disease**	19 (11.2)	1 (1.4)	

Continuous variables are presented as median (IQR) and categorical variables are presented as absolute number (frequency in percent). P-values were calculated with the Mann–Whitney U test for continuous variables and the Chi^2^ test for categorical variables. ALT: alanine aminotransferase; AST: aspartate aminotransferase; AP: alkaline phosphatase; yGT: gamma-glutamyl transferase; IgM: immunoglobulin M; IgG: immunoglobulin G; F-actin: filamentous actin; AIH: autoimmune hepatitis; DILI: drug-induced liver injury; PBC: primary biliary cholangitis; PSC: primary sclerosing cholangitis.

**Table 3:** Demographic and clinical features of the cohort ELISA cut-off 30.

Variable	F-actin ELISA cut-off 30 units		
	F-actin negative by ELISA	F-actin positive by ELISA	p
**n**	**189**	**52**	
**Age [years]**	54.00 [39.00, 63.00]	53.50 [35.00, 62.50]	0.945
**Sex = male (%)**	101 (53.7)	21 (40.4)	0.122
**ALT [U/l]**	40.00 [25.00, 82.00]	58.00 [32.00, 148.50]	0.048
**AST [U/l]**	36.00 [26.00, 74.00]	58.00 [33.00, 188.00]	0.003
**AP [U/l]**	115.00 [72.50, 183.00]	130.00 [106.00, 214.50]	0.032
**yGT [U/l]**	69.00 [30.00, 168.00]	108.00 [41.00, 209.50]	0.147
**Bilirubin [µmol/l]**	12.00 [7.25, 27.75]	22.00 [8.00, 58.00]	0.076
**Albumin [g/l]**	40.00 [34.00, 44.00]	37.00 [27.00, 41.00]	0.007
**IgM [g/l]**	1.23 [0.78, 2.01]	1.57 [1.12, 2.24]	0.031
**IgG [g/l]**	12.55 [10.04, 15.35]	16.20 [13.18, 21.46]	<0.001
**Diagnoses (%)**			0.005
**AIH**	8 (4.2)	11 (21.2)	
**Drug-induced autoimmune-like hepatitis**	1 (0.5)	0 (0.0)	
**Cryptogenic liver disease**	31 (16.4)	7 (13.5)	
**DILI**	5 (2.6)	0 (0.0)	
**Patients after liver transplantation**	8 (4.2)	2 (3.8)	
**Non-autoimmune liver disease**	85 (45.0)	15 (28.8)	
**PBC**	10 (5.3)	6 (11.5)	
**PBC/AIH overlap**	2 (1.1)	2 (3.8)	
**PSC**	10 (5.3)	3 (5.8)	
**PSC/AIH overlap**	2 (1.1)	1 (1.9)	
**Systemic autoimmune disease with liver involvement**	2 (1.1)	1 (1.9)	
**Systemic autoimmune disease without liver involvement**	6 (3.2)	3 (5.8)	
**No liver or systemic autoimmune disease**	19 (10.1)	1 (1.9)	

Continuous variables are presented as median (IQR) and categorical variables are presented as absolute number (frequency in percent). P-values were calculated with the Mann–Whitney U test for continuous variables and the Chi^2^ test for categorical variables. ALT: alanine aminotransferase; AST: aspartate aminotransferase; AP: alkaline phosphatase; yGT: gamma-glutamyl transferase; IgM: immunoglobulin M; IgG: immunoglobulin G; F-actin: filamentous actin; AIH: autoimmune hepatitis; DILI: drug-induced liver injury; PBC: primary biliary cholangitis; PSC: primary sclerosing cholangitis.

### Comparison of IFT and ELISA

**Fig 2:**
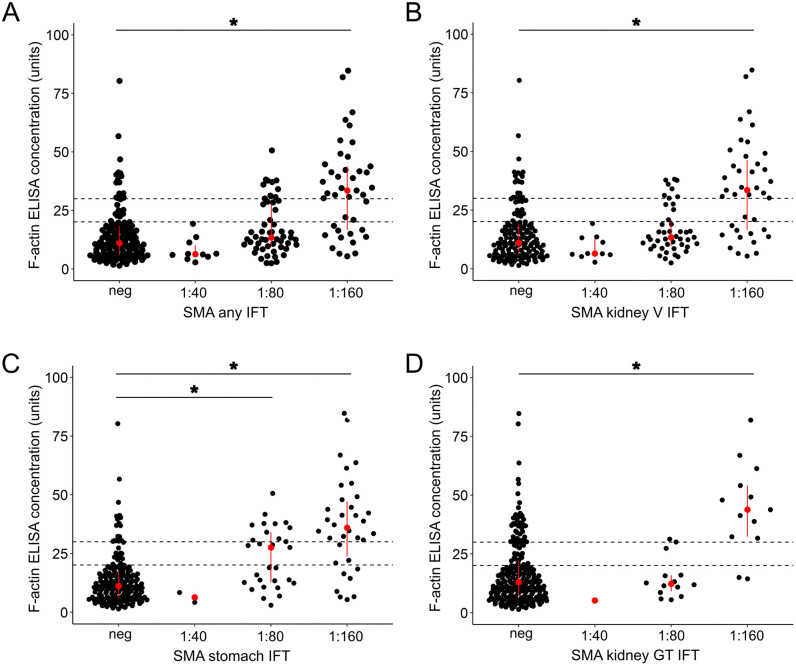
Dot plot of F-actin levels assessed by ELISA with regard to SMA titer on immunofluorescence testing. Each dot represents the level of F-actin antibodies measured by ELISA in units. SMA titers by immunofluorescence testing (IFT) range from negative to 1:160 **(A)** for any SMA staining pattern; **(B)** SMA kidney vessel (V) staining pattern; **(C)** SMA stomach muscularis mucosae staining pattern; **(D)** SMA kidney glomerular (G) and/or tubular (T) staining pattern. Median with first and third quartiles are displayed in red. Dashed lines depict the manufacturer-proposed ELISA cut-offs of 20 and 30 units. Significance was tested using the Wilcoxon rank sum test with Benjamini-Hochberg correction. P-values are displayed for comparisons between negative IFT results and various IFT titer levels, * = p-value of < 0.05.

While there is no difference in F-actin ELISA concentrations between IFT titer 1:40 and negative samples, with higher SMA IFT titers (1:80 and 1:160) the F-actin ELISA concentration median increases continuously (Fig 2A).

When considering the different SMA staining pattens the correlation was found most prominent in stomach SMA staining, with higher F-actin ELISA concentrations at titers of 1:80 and 1:160. ELISA concentration in kidney SMA staining pattens started to increase significantly at a titer of 1:160 (Fig 2B-D).

Moving forward we used IFT titer ≥1:80 as the cut-off for comparison analysis.

**Fig 3:**
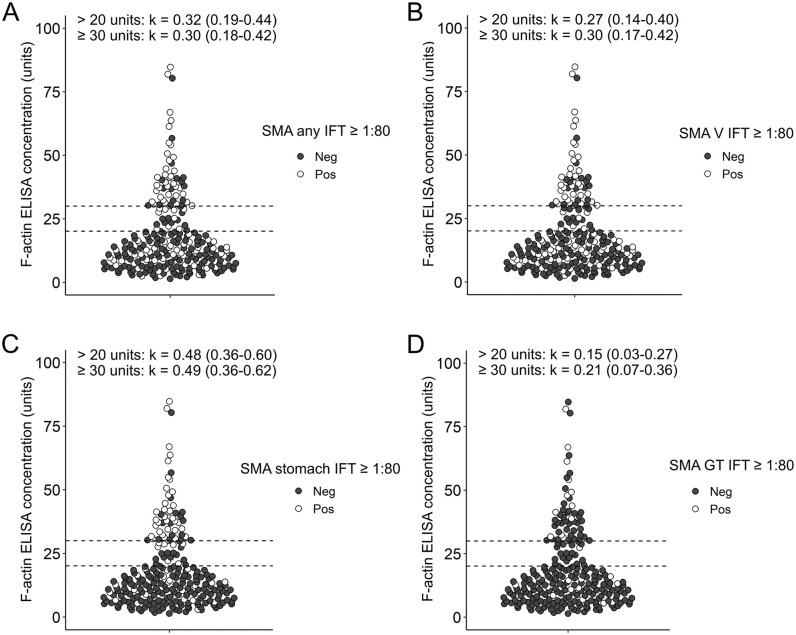
Dot plot of F-actin levels assessed by ELISA with regard to presence of SMA on immunofluorescence testing. Each dot represents the level of F-actin antibodies measured by ELISA in units. White dots were positive for SMA by immunofluorescence testing (IFT) and black dots were negative at a cut-off titer of 1:80. **(A)** for any SMA staining pattern; **(B)** SMA kidney vessel (V) staining pattern; **(C)** SMA stomach muscularis mucosae staining pattern; **(D)** SMA kidney glomerular (G) and/or tubular (T) staining pattern. Cohen’s kappa (k) is presented on top of the figure for the manufacturer-proposed cut-offs of 20 and 30 units respectively with the corresponding 95% confidence interval. Dashed lines depict cut-offs of 20 and 30 units.

Agreement between SMA IFT and the commercial F-actin ELISA using the cut-off of 20 units and 30 units as recommended by the manufacturer was slight for any SMA (total SMA positivity regardless of the staining patten) and kidney SMA pattens (Fig 3A, B, D).

SMA staining of kidney vessels and subsequently any SMA staining showed a high number of false-negative ELISA samples leading to low agreement between both tests. SMA glomeruli and tubules staining showed false-negatives and false-positives at both cut-offs.

SMA stomach staining showed the highest agreement (moderate agreement), with kappa values of 0.48 (0.36–0.60) and 0.49 (0.36–0.62) respectively (Fig 3C).

Further subset analysis of the SMA stomach staining pattern revealed slightly higher kappa values in AIH and other autoimmune diseases than in the subset of samples without an autoimmune disease (S1 Fig).

Moderate agreement related mostly to false-negative results in comparison to IFT at an ELISA cut-off of 30 units and to both false-negatives and false-positives at an ELISA cut-off of 20 units. Sensitivities were 66.7% and 55.5% for ELISA cut-off of 20 and 30 units, respectively. Specificities reached 83.7% and 90.4%, respectively for both ELISA cut-offs (S1 Table).

For cut-off optimization we used SMA stomach staining (cut-off ≥ 1:80) as a reference since it showed the highest agreement with F-actin ELISA concentrations of all SMA staining patterns.

**Fig 4:**
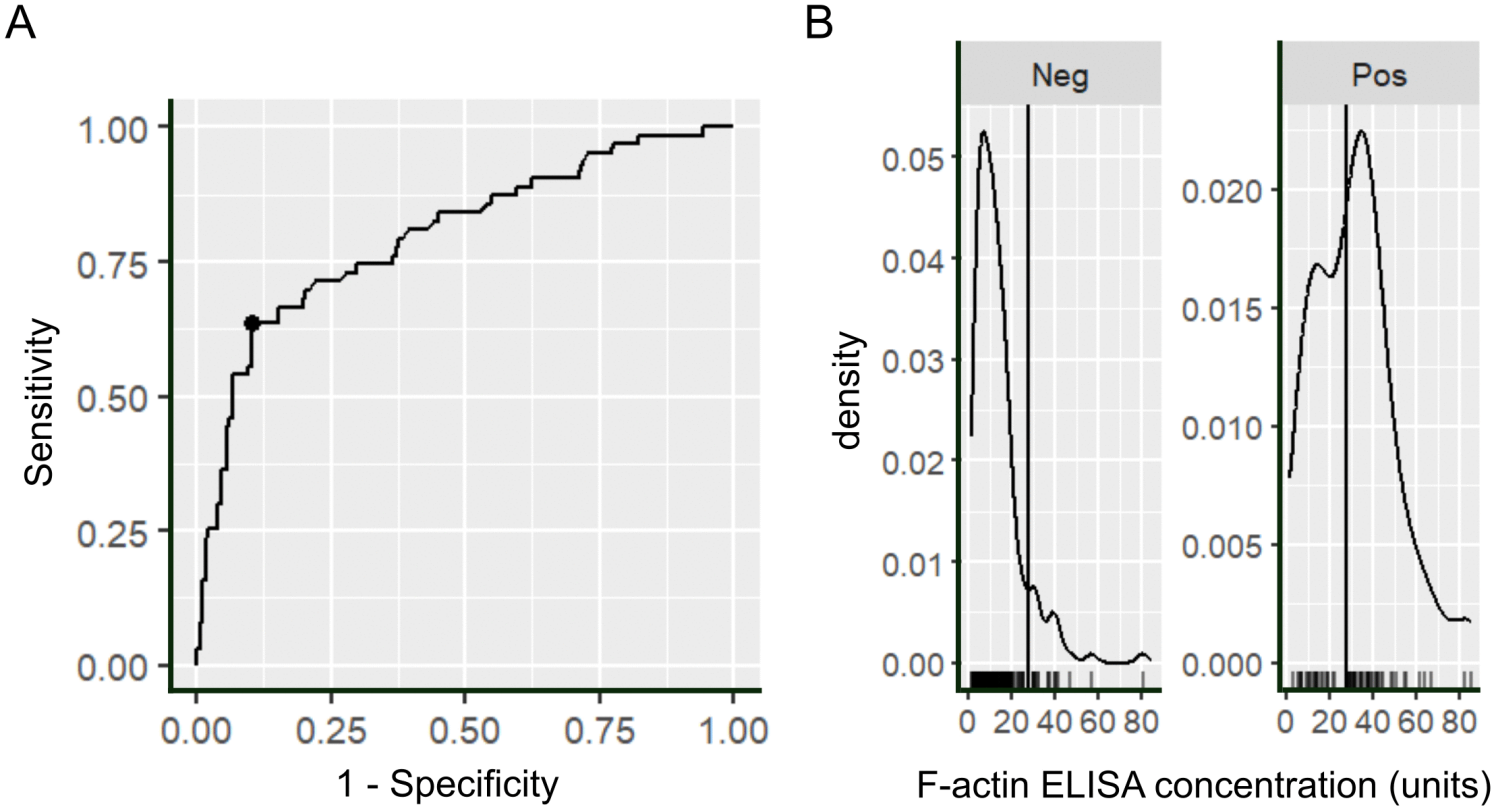
Optimization of the ELISA cut-off with AUC and the Youden index. **(A)** Receiver operating curve of the F-actin ELISA with SMA assessment by IFT on rodent stomach with a cut-off of 1:80 as the reference standard. **(B)** distribution of F-actin levels in units in IFT-negative (Neg) and IFT-positive samples (Pos) assessed by IFT on rodent stomach.

We used an AUROC and Youden index to calculate an optimized cut-off for the commercial ELISA (Fig 4A).

**Fig 5:**
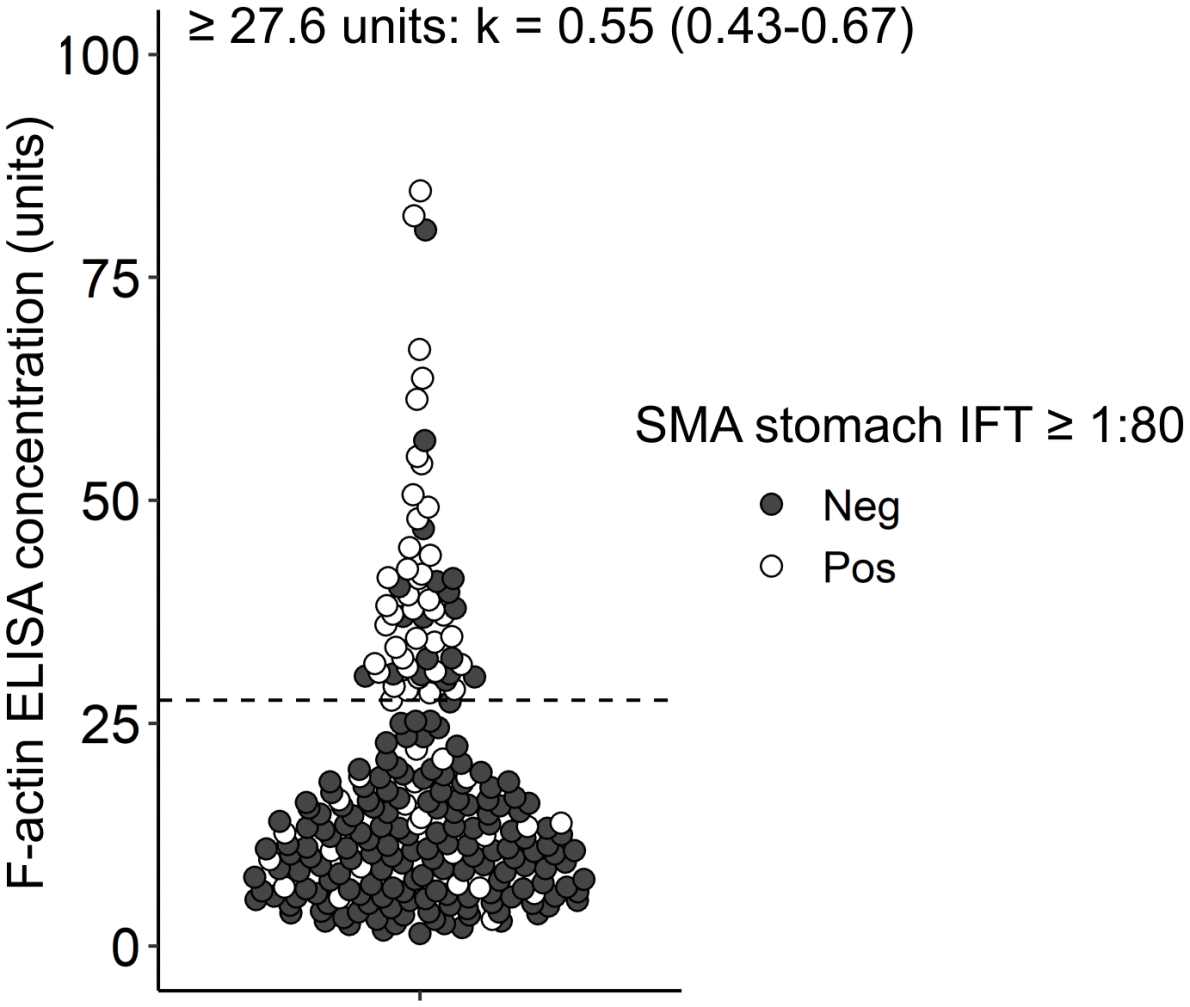
Dot plot of F-actin levels assessed by ELISA with optimized cut-off to predict presence of stomach SMA on immunofluorescence testing. Each dot represents the level of F-actin antibodies measured by ELISA in units. White dots were positive for SMA stomach by immunofluorescence testing (IFT) and black dots were negative at a cut-off of 1:80. Cohen’s kappa (k) is presented on top of the figure for the cut-off optimized by the Youden index with the corresponding 95% confidence interval. Dashed line depicts optimized cut-off of 27.6 units.

The optimized Youden index revealed 27.6 units as the best cut-off to maximize agreement between IFT and ELISA with an AUC of 0.8. Fig 4B depicts a density blot of F-actin ELISA concentrations in IFT stomach positive and negative samples using the optimized cut-off to determine F-actin ELISA positivity. By adjusting the cut-off to 27.6 units kappa was slightly increased with a kappa value of 0.55 (0.43–0.67) (Fig 5) mainly by reducing the number of false-positives in comparison to the ELISA cut-off of 20 units. At the optimized cut-off false-positives were comparable to the manufacturer-proposed cut-off of 30 units in the ELISA with less false-negatives. Sensitivity reached 63.4% and specificity 89.9% using the optimized cut-off (S2 Table).

## Discussion

In this study we show that in real-world clinical practice results from F-actin ELISA and SMA assessed by IFT on rodent tissue reach only slight to moderate agreement. There were considerably differences in reliability depending on the SMA staining pattern. SMA staining of the stomach muscularis mucosae showed the highest agreement with F-actin ELISA concentrations, while the kidney staining patterns only showed low agreement. F-actin ELISA concentrations increased with increasing SMA titers regardless of the staining pattern. While international guidelines recognize SMA titers ≥1:40 as a positive SMA signal, different studies emphasize the importance of local validation and local cut-off adjustment [2,4,20]. In our centre a SMA titer of ≥1:80 has been identified as the optimized cut-off [20]. In line with these findings ELISA concentrations did not vary between SMA negative and SMA 1:40, therefore we used a SMA titer of ≥1:80 as a local cut-off for further agreement analyses. Since the highest inter-rater reliability was demonstrated between SMA stomach staining and F-actin ELISA concentration we used the stomach SMA for further cut-off adjustment. By means of the Youden index the calculated optimized cut-off was located between the two cut-offs suggested by the manufacturer and was able to increase the kappa value slightly. Sensitivity and specificity were also optimized slightly using the cut-off 27.6 units. All in all, these are minor, probably not clinically meaningful improvements to the concordance between SMA IFT and the F-actin ELISA, which remains moderate. These results suggest that SMA IFT and F-actin ELISA are not interchangeable tests in the work up of AILD but rather complementary and should be used as such. To date, there has not been any study investigating the agreement between an ELISA detecting F-Actin and IFT for the detection of SMA on rodent tissue substrates as typically used for autoantibody detection in case of suspicion for AILD in an unbiased setting [7,14]. However, one study found a good correlation between ELISA and IFT in pre-selected SMA-positive AIH cases [21].

Other studies comparing F-actin ELISA with the presence of IFT on SMA determined higher optimized cut-off values for the F-actin ELISA [21–23]. Three main differences can be found when comparing the design of published studies and ours. First, these studies used AIH enriched cohorts or even SMA-positive pre-selected AIH cases, while our study included all serum samples that were tested during the study period in an unbiased fashion with regard to disease and presence of autoantibodies. SMA is known to be enriched in patients with AIH but also occurs in various other liver diseases as well as unspecifically [5]. Our subgroup analysis demonstrated higher agreement between the two tests when including only autoimmune diseases and more specifically samples from patients with AIH thereby resembling published results more closely (S1 Fig).

Second, while not specified by Villatta et al. and Frenzel et al., Granito et al., used serum exclusively from untreated AIH patients. Our study included samples regardless of patients’ treatment status. Autoantibodies are known to change, namely disappear and reappear, in longitudinal studies of AIH patients [24]. Whether this is a predictor of treatment success is unclear. Unfortunately, our study lacked sufficient sample size of treated AIH patients or even longitudinal samples of the same patients to assess the presence of SMA and F-Actin autoantibodies with regard to treatment efficacy.

Third, at least two studies only used kidney glomerular (G) and kidney tubular (T) SMA patterns [22,23]. In contrast to the published results kidney staining patterns GT showed a low concordance with the F-actin ELISA concentration in our study. These inconsistent results may again be due to differences in the study population. At this point F-actin antigen specificity of SMA patterns remains controversial. Kidney GT patterns have been shown to be highly specific, while not highly sensitive, for AIH diagnosis [14]. Some studies suggest this to be due to F-actin antigen specificity, while others could show non-F-actin antigens responsible for kidney GT SMA patterns of AIH patients using inhibition studies [25]. F-actin has also been identified as the main antigenic target of SMA in rodent stomach using inhibition studies [10].

Further studies are needed to determine antigen specificity of different SMA staining patterns and their diagnostic relevance in the context of evaluating a patient for an AILD-origin of liver injury.

Regardless of the SMA staining pattern on IFT F-actin is not the only antigenic target of SMA. SMA also targets other proteins found in smooth muscles, e.g., tubulin, vimentin, desmin, and skeletin [6,26]. Therefore disagreement between both tests with negative F-actin ELISA and positive SMA stomach IFT is most likely due to non-actin SMAs. Likewise, limited overlap between IFT and more antigen specific ELISAs in the setting of liver diseases has also been reported for ANA [4].

A limitation is the single centre design of our study. However, it resembles real world practice as all patient samples that were sent in a three-months period and their respective clinically validated results were used for the analysis thereby excluding selection bias. It is important to note that IFT is susceptible to inter-laboratory variability and results from our monocentric study might not transfer directly to other centres. Local validation and verifications in different cohorts are necessary to find optimized local cut-offs and assess generalizability of our findings.

In real-life observations autoimmune liver diseases only account for a small number of cases in the work up of liver injuries. In our study, in most cases a non-autoimmune liver disease was diagnosed. It is important to note that the strength of this study is the comparative performance demonstration in a real-world laboratory scenario comparing the ELISA- and IFT-based detection of F-actin. With only 26 AIH patients (including all subtypes and overlap patients) this study is neither powered to assess the diagnostic accuracy of SMA staining patterns or the F-actin ELISA to predict AIH nor to analyse of the presence of any of the two antibodies associates with treatment response or disease activity. Since F-actin specific antibodies have been described to be highly specific for AIH the ELISA might help to differentiate different types of SMAs [10,27], especially as F-actin SMA was correlated with disease behaviour in patients with AIH [28–30]. However, while demonstrating excellent accuracy in the context of liver disease [4] a recent study highlighted presence of F-actin SMA especially in rheumatological diseases [31].

Therefore, our study emphasizes the context-sensitive interpretation of any SMA test carried out for the work-up of a specific patient`s disease. It further strengthens recent findings that highlight that those diagnostic cut-offs proposed for the diagnosis of AIH for autoantibody assessment may not be universally applicable across all centres with sensitivities varying among them and there is a need to locally adapt cut-offs to maintain high diagnostic accuracy [4,18].

In conclusion our study, in contrast to earlier publications, shows that positive F-actin ELISA results mostly correlated with positive stomach SMA staining’s. However, even after cut-off optimization agreement remains moderate. The F-actin ELISA is therefore not able to predict SMA positivity sufficiently in clinical practice. To evaluate its benefit as a screening tool for AILD, independently from SMA screenings by IFT, future multicentre studies are needed.

## Supporting information

S1 Fig Dot blot of F-actin levels assessed by ELISA with regard to presence of stomach SMA on immunofluorescence testing for different diagnosis subgroups. Each dot represents the level of F-actin antibodies measured by ELISA in units. White dots were positive for SMA stomach by immunofluorescence testing (IFT) and black dots were negative at a cut-off titer of 1:80. Cohen’s kappa (k) is presented on top of the figure for the manufacturer-proposed cut-offs of 20 and 30 units respectively with the corresponding 95% confidence interval **(A)** patients diagnosed with an autoimmune disease other than AIH; **(B)** patients diagnosed with AIH; **(C)** patients without a liver or autoimmune disease; **(D)** patients diagnosed with a non-autoimmune liver disease. Dashed lines depict F-Actin ELISA cut-offs of 20 and 30 units.
(TIFF)

S1 Table Crosstable of ELISA and IFT stomach results at manufacturer-proposed cut-offs. (DOCX)

S2 Table Crosstable ELISA and IFT results at the optimized cut-off using the Youden index. (DOCX)

## Abbreviations

AIH: Autoimmune Hepatitis
AILD: Autoimmune liver disease
ALT: Alanine aminotransferase
AST: Aspartate aminotransferase
ANA: Antinuclear antibody
AP: Alkaline phosphatase
DILI: Drug-induced liver injury
F-actin: Filamentous actin
G: Glomerular
G-actin: Globular actin
yGT: Gamma-glutamyl transferase
IFT: Immunofluorescence testing
IgM: Immunoglobulin M
IgG: Immunoglobulin G
LC1: Liver cytosol type 1 antibody
LKM: Liver-kidney microsomal antibody
LP: Liver pancreas antibody
MHH: Hannover Medical School
NPV: Negative predictive value
PBC: Primary Biliary Cholangitis
PSC: Primary sclerosing cholangitis
PPV: Positive predictive value
PSC: Primary Sclerosing Cholangitis
SLA: Soluble-liver-antigen antibody
SMA: Smooth muscle antibody
T: Tubular
V: Vessel
