# Supplementary figures and images for: Comparative evaluation of immunoserological detection of F-actin antibodies
Source: PLoS One. 2026 Apr 7;21(4):e0345250. doi: 10.1371/journal.pone.0345250 (PMC13056164; doi:10.1371/journal.pone.0345250)

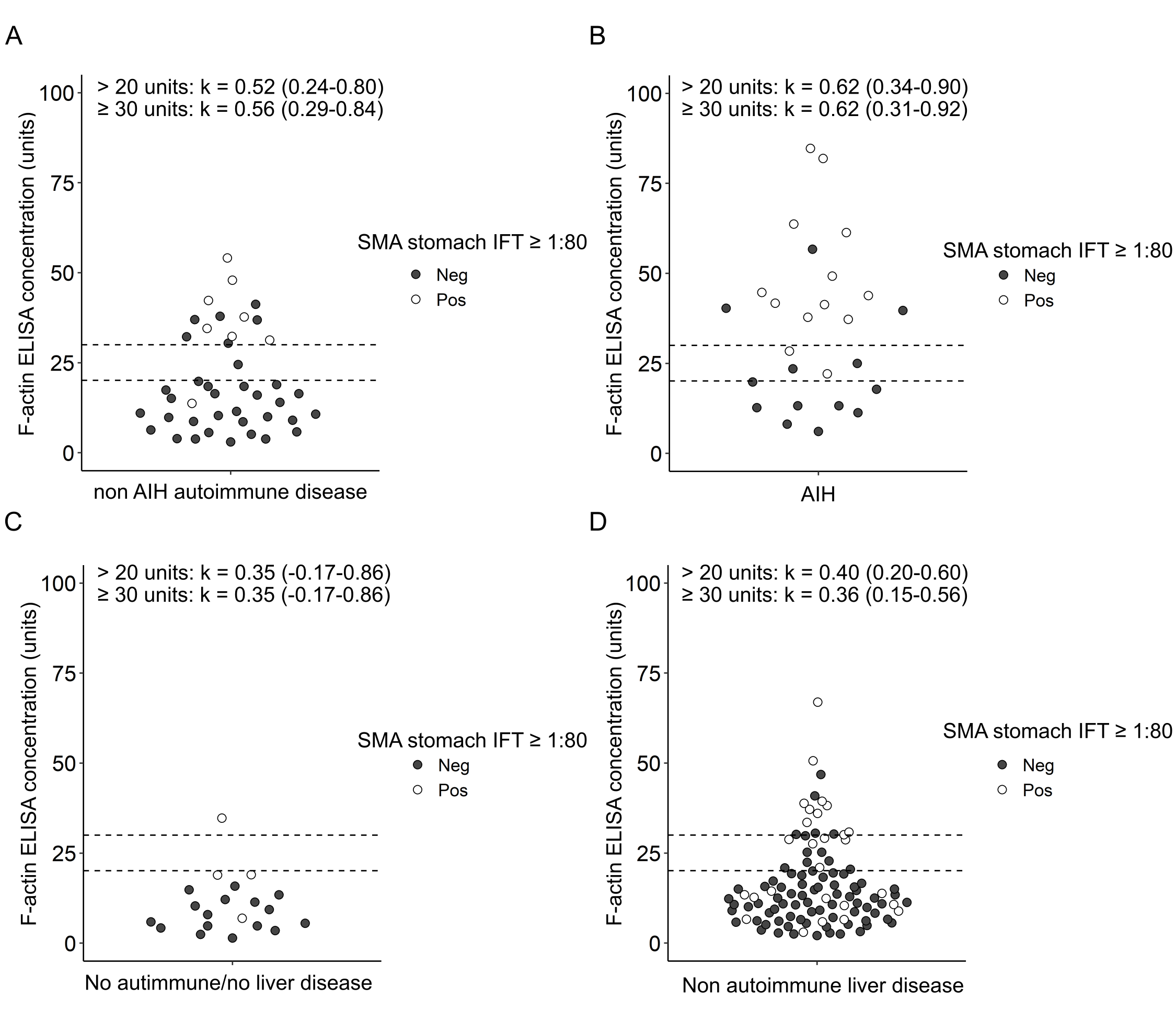

Supplement: S1 Fig — Each dot represents the level of F-actin antibodies measured by ELISA in units. White dots were positive for SMA stomach by immunofluorescence testing (IFT) and black dots were negative at a cut-off titer of 1:80. Cohen’s kappa (k) is presented on top of the figure for the manufacturer-proposed cut-offs of 20 and 30 units respectively with the corresponding 95% confidence interval (A) patients diagnosed with an autoimmune disease other than AIH; (B) patients diagnosed with AIH; (C) patients without a liver or autoimmune disease; (D) patients diagnosed with a non-autoimmune liver disease. Dashed lines depict F-Actin ELISA cut-offs of 20 and 30 units. (TIFF) [file pone.0345250.s001.tiff]
