# Supplementary material for: Comparative evaluation of immunoserological detection of F-actin antibodies
Source: PLoS One. 2026 Apr 7;21(4):e0345250. doi: 10.1371/journal.pone.0345250 (PMC13056164; doi:10.1371/journal.pone.0345250)
Supplement: S1 Table — (DOCX) [file pone.0345250.s002.docx]

**S1 Table. Crosstable of ELISA and IFT stomach results at manufacturer-proposed cut-offs**

| **ELISA Cut-off 20 units** | **IFT stomach positive** | **IFT stomach negative** |  |  |
| --- | --- | --- | --- | --- |
| **ELISA positive** | 42 | 29 | 59.2 % | PPV |
| **ELISA negative** | 21 | 149 | 87.6 % | NPV |
|  | 66.7 % | 83.7 % |  |  |
|  | sensitivity | specificity |  |  |
| **Optimized ELISA cut-off 30 units** | **IFT stomach positive** | **IFT stomach negative** |  |  |
| **ELISA positive** | 35 | 17 | 67.3 % | PPV |
| **ELISA negative** | 28 | 161 | 85.2 % | NPV |
|  | 55.5 % | 90.4 % |  |  |
|  | sensitivity | specificity |  |  |

IFT: immunofluorescence testing. IFT cut-off ≥ 1:80, PPV: Positive Predictive Value, NPV: Negative Predictive Value
