# Supplementary material for: Comparative evaluation of immunoserological detection of F-actin antibodies
Source: PLoS One. 2026 Apr 7;21(4):e0345250. doi: 10.1371/journal.pone.0345250 (PMC13056164; doi:10.1371/journal.pone.0345250)
Supplement: S2 Table — (DOCX) [file pone.0345250.s003.docx]

**S2 Table. Crosstable ELISA and IFT results at the optimized cut-off using the Youden index**

| **Optimized ELISA cut-off 27.6 units** | **IFT stomach positive** | **IFT stomach negative** |  |  |
| --- | --- | --- | --- | --- |
| **ELISA positive** | 40 | 18 | 69.0 % | PPV |
| **ELISA negative** | 23 | 160 | 87.4 % | NPV |
|  | 63.4 % | 89.9 % |  |  |
|  | sensitivity | specificity |  |  |

IFT: immunofluorescence testing. IFT cut-off ≥ 1:80, PPV: Positive Predictive Value, NPV: Negative Predictive Value
